# Effects of oilseed rape green manure on phosphorus availability of red soil and rice yield in rice–green manure rotation system

**DOI:** 10.3389/fpls.2024.1417504

**Published:** 2024-06-14

**Authors:** Chi-ming Gu, Yin-shui Li, Lu Yang, Jing Dai, Wenshi Hu, Chang-bing Yu, Margot Brooks, Xing Liao, Lu Qin

**Affiliations:** ^1^ Oil Crops Research Institute of the Chinese Academy of Agricultural Sciences, Key Laboratory of Biology and Genetic Improvement of Oil Crops Ministry of Agriculture of China, Wuhan, Hubei, China; ^2^ Department of Biochemistry and Microbiology, Rhodes University, Grahamstown, South Africa

**Keywords:** oilseed rape green manure, phosphorus availability, soil phosphorus components, organic farming, rice production

## Abstract

Improving the nutrient content of red soils in southern China is a priority for efficient rice production there. To assess the effectiveness of oilseed rape as green manure for the improvement of soil phosphorus nutrient supply and rice yield in red soil areas, a long-term field plot experiment was conducted comparing two species of rape, *Brassica napus* (BN) and *Brassica juncea* (BJ). The effects of returning oilseed rape on soil phosphorus availability, phosphorus absorption, and yield of subsequent rice under rice–green manure rotation mode were analyzed, using data from the seasons of 2020 to 2021. The study found that compared with winter fallow treatment (WT) and no-tillage treatment (NT), the soil available phosphorus content of BN was increased, and that of BJ was significantly increased. The content of water-soluble inorganic phosphorus of BJ increased, and that of BN increased substantially. Compared with the WT, the soil organic matter content and soil total phosphorus content of BN significantly increased, as did the soil available potassium content of BJ, and the soil total phosphorus content of BJ was significantly increased compared with NT. The soil particulate phosphorus content of BJ and BN was significantly increased by 14.00% and 16.00%, respectively. Compared with the WT, the phosphorus activation coefficient of BJ was significantly increased by 11.41%. The rice plant tiller number under the green manure returning treatment was significantly increased by 43.16% compared with the winter fallow treatment. The green manure returning measures increased rice grain yield by promoting rice tiller numbers; BN increased rice grain yield by 9.91% and BJ by 11.68%. Based on these results, returning oilseed rape green manure could augment the phosphorus nutrients of red soil and promote phosphorus availability. Rice–oilseed rape green manure rotation could increase rice grain yield.

## 1 Introduction

Insufficient organic fertilizer, the lack of biological fertilization measures, and single planting systems as well as improper use of chemical fertilizer will lead to the decrease of soil organic matter content and deterioration of soil physical and chemical properties and soil structure. The consequence in the arable layer of farmland is the reduction in soil sustainable production capacity (Li et al., 2021). A lack of bioavailable phosphorus in soil restricts rice yield and has become a hot point for rice production research in red soil areas (Xie et al., 2022; Gu et al., 2023). As a high-quality organic fertilizer, green manure can not only effectively enable the utilization of fallow fields but also significantly decrease the soil degradation caused by the long-term application of chemical fertilizers and monocropping (Asghar and Kataoka, 2022). The pressure from demand for staple foods due to increasing population means the area used for summer crops is constantly expanding, especially in rice-growing regions, leaving insufficient time for normal winter crop production. At the same time, great importance is attributed, globally, to the protection of cultivated land and the improvement of arable land quality. Thus, the planting of green manure in winter to fertilize the soil and improve the quality and efficiency of subsequent crops has become a common choice and has attracted more and more attention (Lyu et al., 2024; Ren et al., 2024). Studies have shown that the application of green manure in different rotations has a significant effect on soil physical and chemical properties and the yield of subsequent crops (Gu et al., 2021; Lyu et al., 2024; Ren et al., 2024). Green manure is a general term for the use of plants grown specifically to be plowed back into the soil as fertilizer for subsequent crops. Green manure is a high-quality, clean organic fertilizer that is a renewable biological resource. It has a significant effect on improving and fertilizing soil and can lay a foundation for the high yield and high quality of crops (Xie et al., 2016). Planting green manure can reduce weeds in the field and reduce soil nutrient loss (Yu et al., 2014). Root exudates of green manure crops can enhance soil microbial and soil biochemical activity, dissolve and utilize insoluble phosphorus in the soil, and increase soil available phosphorus levels (Gu et al., 2023; Yuan et al., 2023).

Existing research has shown that oilseed rape as green manure could increase soil organic matter and soil bioavailable phosphorus nutrient content and improve soil fertility. Compared with common leguminous green manure, oilseed rape green manure has the characteristics of wide adaptability and low cost and functions as a biological control (Fu et al., 2012; Srivastava and Ghatak, 2017). In addition, it can increase the total porosity of paddy soil, reduce bulk density, improve soil physical properties, and increase the activity of soil urease and acid phosphatase (Tang et al., 2017). The results of intercropping potato with spring rape as green manure showed that the application of green manure could reduce the application rate of potato fertilizer by 15%–20% without reducing the yield (Jing et al., 2007). After oilseed rape was returned to the field as green manure in another rice–rape rotation study, the leaf area index, flag leaf length, leaf width, chlorophyll content, and photosynthetic rate of subsequent rice plants were increased to a certain extent, and the dry matter accumulation of rice was increased, thereby increasing the subsequent rice yield (Xie et al., 2016). In addition, the surface coverage of green manure crops planted in winter will affect the soil moisture conditions of the farmland and change the activity of soil microorganisms and permeability and redox potential of the soil, affecting the cycle of phosphorus in the soil, thereby affecting the absorption and utilization of phosphorus by subsequent crops (LeBlanc, 2022).

**Figure 1:**
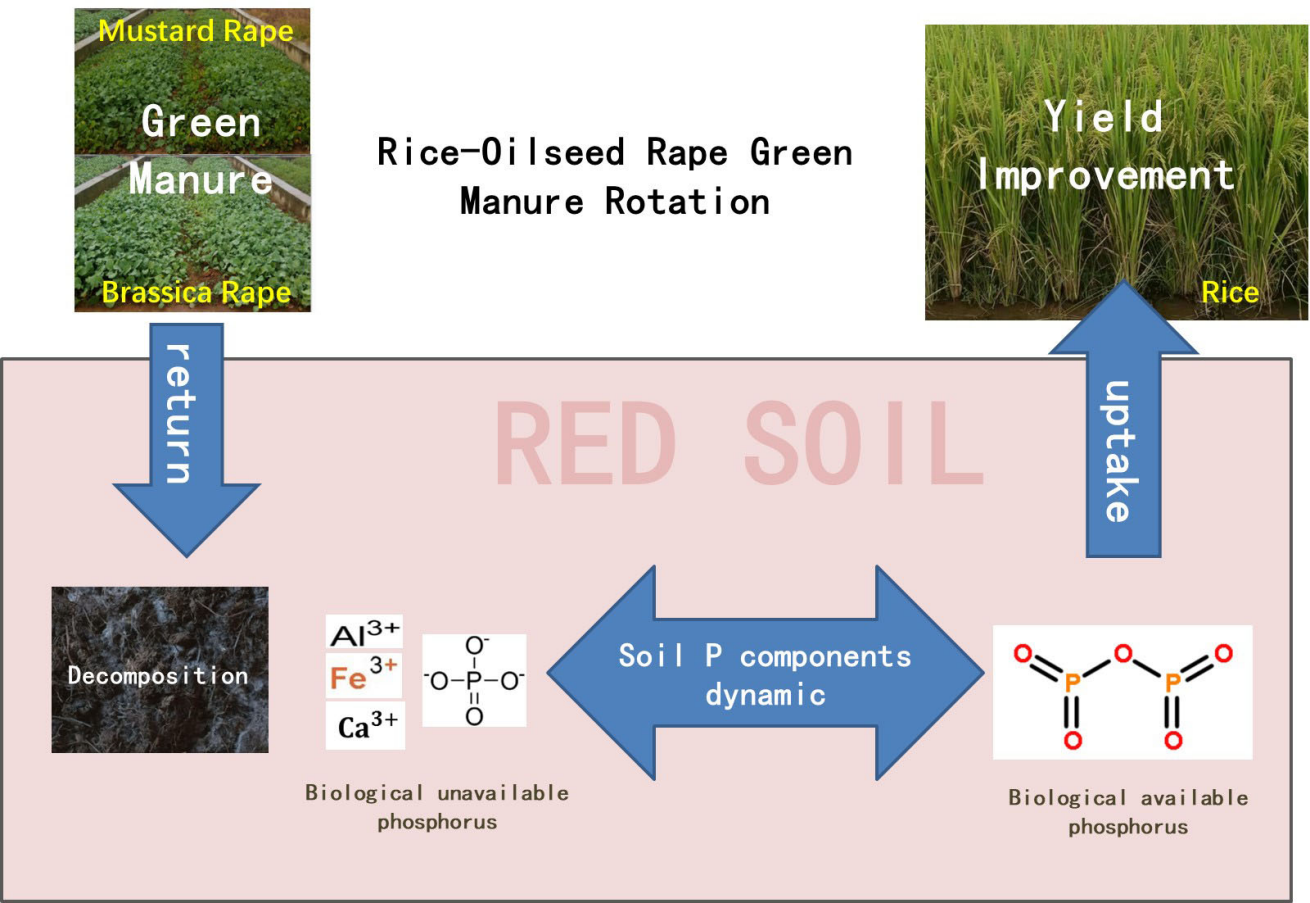
Rice–oilseed rape green manure rotation modes.

However, the current research on rice–oilseed rape rotation is mainly grain-oriented. There are relatively few studies that focus on the effect of oilseed rape green manure returning on soil phosphorus availability and then on rice phosphorus absorption, which are known to restrict the development of green organic sustainable agriculture in red soil, and thus affect rice yield. To analyze and compare the differences in the effect of the application of different species of oilseed rape green manure on soil phosphorus availability, rice phosphorus uptake, and rice yield after returning in rice production, a positioning field experiment was carried out in Zhanggong Town, Jinxian County, Jiangxi Province. The objective of this study is to demonstrate the effects of oilseed rape green manure amendment on the bioactivity of phosphorus and rice yield in the red soil area, providing practical bases for both the improvement of paddy soil quality and green production of rice by the application of oilseed rape green manure (**Figure 1**).

## 2 Materials and methods

### 2.1 Experimental site

This study was initiated in September 2017 at the field experimental base of the Chinese Academy of Agricultural Science in Hongrang Village, Zhanggong Town, Jinxian City, Jiangxi Province, China. The base is at 28°21′10″N, 116°11′6″E and has an altitude of 26.7 m. It has a typical humid subtropical monsoon climate, with abundant water and heat resources. The rainfall is mainly concentrated from April to August. The annual average rainfall is 1,858 mm, the annual average evaporation is 1,150 mm, the annual average temperature is 17.92°C, the annual effective radiation is 660.42 MJ m^−2^, and the frost-free period is 262 days. The soil texture is sandy loam with the following chemical characteristics: soil total organic matter (SOM) was 17.42 g kg^−1^, pH (H_2_O:soil = 5:1) was 5.34, alkali-hydrolyzable nitrogen (AN) was 98.98 mg kg^−1^, available phosphorus (AP) was 6.50 mg kg^−1^, and available potassium (AK) was 55.53 mg kg^−1^. Rice is one of the primary crops in the region, with Jiangxi Province being one of the main red soil areas of China. Data in this manuscript were collected from October 2020 to September 2021.

### 2.2 Experimental design and field management

The experimental design was based on the random block design. The planting mode was rice (*Oryza sativa* L., variety name: Shenliangyou 5814)–green manure rotation, with four treatments: brassica rape (*Brassica napus* L., BN) (variety name: Zhongyoufei No. 1), mustard rape (*Brassica juncea*, BJ) (variety name: Zhongjieyou No. 1), winter fallow (WT), and no tillage (NT), with three replicates each, spanning a total of 12 plots. All the green manure was sown in the second week of October of each year. The aboveground biomass was cut to 10–20 cm at the stage of flowering in the middle of March and returned into the 15–30-cm soil layer by grubbing and using a rotary tiller machine. Rice was sown 25 days before green manure return at a seedling bed in another field. Ten days after green manure incorporation, the field was plowed, irrigated, and soaked to prepare for rice transplanting. Then, rice seedlings were transplanted into each plot with a density of 30,000 plants per hectare and harvested in the middle of September. The chemical fertilizer used in the experiment comprised urea (46% N), superphosphate (12% P_2_O_5_), and potassium oxide (60% K_2_O). An equal amount of fertilizer was applied as base fertilizer in all plots in the green manure season (N:P_2_O_5_:K_2_O was 15:0:0 kg ha^−1^). In the rice season, the application rate of N, P_2_O_5_, and K_2_O fertilizer in all plots was 195, 90, and 135 kg ha^−1^, respectively. The full standard quantities of phosphorus and potassium fertilizers of the rice season were used as base fertilizer, while 50% of standard nitrogen fertilizer was used as base fertilizer, with 20% applied as topdressing in the tillering stage and 30% applied as topdressing in the earing stage. In the green manure season, irrigation was done after sowing and artificial weed control was performed during the seedling stage. In the rice season, herbicides were sprayed after plowing, and irrigation and drainage were carried out at the tillering and ear-filling stages of rice, respectively. During the growth periods of green manure and rice, pest prevention and control measures were carried out as necessary.

### 2.3 Sampling and analysis

Before green manure returning, three representative areas of 0.5 m^2^ were chosen in each plot and cut level with the ground in order to weigh the yield of green manure and sample for nutrient testing at the same time. At the rice tillering stage, three representative 0.5 m^2^ quadrats were selected to investigate the tillering of rice. Rice plant samples were taken during the harvest stage for yield calculation and nutrient testing. All plant samples were separated into leaf, seed, or root, after which they were air-dried, ground, sieved, and then digested with H_2_SO_4_-H_2_O_2_ for nutrient testing. For plant nutrient testing, the total nitrogen (TN) concentration of the samples was determined by the Kjeldahl method, and total phosphorus (TP) and total potassium (TK) were tested by ICP (Germany) after extraction by heating digestion with H_2_SO_4_-H_2_O_2_ (Bao, 2000).

Mixed soil samples were collected right before the fertilizer application when rice was transplanted. They were taken from the soil surface layer (0–20 cm) from 5 points in each plot using a soil borer. After removing animal and plant residues and pebbles, the soil samples were air-dried and put through a 100- or 200-mesh sieve for further analysis. SOM was measured by the K_2_Cr_2_O_7_ oxidation method, available N (AN) was measured by the alkaline diffusion method, available phosphorus (AP) concentration of the samples was determined by the Olsen method after extraction with 0.03 mol L^−1^ of NH_4_F and 0.025 mol L^−1^ of HCl, available K (AK) was measured by flame photometry after extraction with 1 mol L^−1^ of NH_4_OAc, and pH (1:10, soil to water rate) was determined with a pH meter (Bao, 2000). For soil total phosphorus (STP), dry soil (2 g) was heated with 0.2 g of sodium hydroxide at 720°C for 20 min. After cooling, the soil was washed with distilled water. Then, 10 mL of 4.5 M H_2_SO_4_ was added to the combined liquids and made up to 100 mL by adding distilled water. The extract was used for spectrophotometric analysis (Bao, 2000). Total water-soluble phosphorus (TWSP) was determined by placing 2 g of dry soil sample into 20 mL of distilled water, oscillating under 250 r min^−1^ for 1 h, filtering through a 0.45-μm filter, and digesting the filtrate with potassium persulfate. The extract was used for spectrophotometric analysis. Soil total phosphorus (STP) minus total water-soluble phosphorus (TWSP) equaled to particulate phosphorus (PP). Water-soluble inorganic phosphorus (WSIP) was determined by placing 2 g of dry soil sample into 20 mL of distilled water, oscillating under 250 r min^−1^ for 1 h, filtering through a 0.45-μm filter, and then using the extract for spectrophotometric analysis (Zhu, 2011). TWSP minus WSIP equaled to water-soluble organic phosphorus (WSOP). Nutrient accumulation of plants (NA) was calculated using the following equation:


$$\text{NA}={\text{W}}_{\text{L}}\times {\text{N}}_{\text{L}}+{\text{W}}_{\text{S}}\times {\text{N}}_{\text{S}}+{\text{W}}_{\text{R}}\times {\text{N}}_{\text{R}}$$

where *W*_L_ is the weight of the leaf, *W*_S_ is the weight of the seed, *W*_R_ is the weight of the roots, *N*_L_ is the nutrient content of the leaf, *N*_S_ is the nutrient content of the seed, *N*_R_ is the phosphorus content of the roots, and NA is the nutrient accumulation.

Total phosphorus and available phosphorus are two important indicators to measure the state of soil phosphorus. The ratio of available phosphorus to total phosphorus is expressed as the soil phosphorus activation coefficient (PAC), which can be used as a relative indicator to characterize the effectiveness of soil phosphorus. PAC was calculated using the following equation:


$$\text{PAC}={\text{C}}_{\text{AP}}/{\text{C}}_{\text{TP}}\times 100\%$$

where *C*_AP_ is the content of soil available phosphorus, *C*_TP_ is the content of soil total phosphorus, and PAC is the activation coefficient of soil phosphorus.

### 2.4 Statistical analysis

All data collected were statistically analyzed as a completely randomized design using ANOVA to test the differences in grain yield, soil nutrient contents, yield, and yield components among treatments. Analysis of variance (ANOVA) was carried out to determine the differences between the measured parameters for different treatments. The least significant difference (LSD) at *p* = 0.05 was used to elucidate any significant differences. All analyses were conducted using SPSS statistical software (ver. 11.0, SPSS, Chicago, IL, USA).

## 3 Results

### 3.1 Differences between yield of oilseed rape green manure species

**Figure 2:**
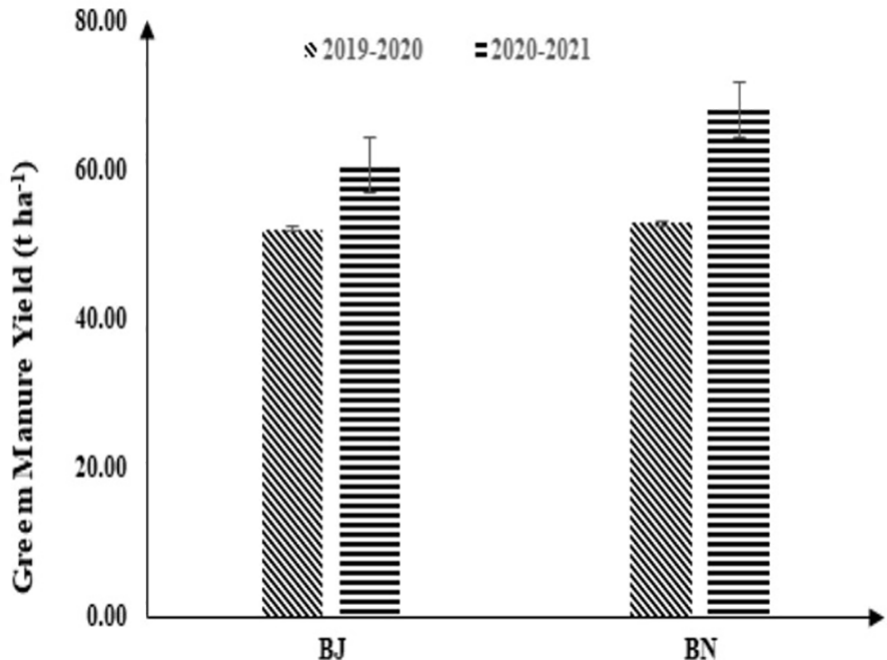
Comparison of oilseed rape green manure yield over two growth seasons. BJ represents mustard oilseed rape green manure, and BN represents brassica oilseed rape green manure.

The difference in yield of different oilseed rape green manure species is shown in **Figure 2**. Comparing the yield of two types of green manure rape, it was found that the yield of BN green manure rape was higher than that of BJ. The average fresh plant yields of BJ and BN in the two species were 56.36 and 60.35 t ha^−1^, respectively.

The accumulation of nitrogen, phosphorus, and potassium nutrients in different oilseed rape green manure is shown in **Table 1**. It was found that the amount of nitrogen and phosphorus accumulation of BN was significantly higher than that in BJ, with an average increase of 43.52% and 23.86%, respectively. There was no significant difference in the cumulative amount of potassium between the two species.

**Table 1:** Differences in nutrient accumulation of green manure in different rotation modes (kg ha^−1^).

	Treatments	Nitrogen returning	Phosphorus returning	Potassium returning
2019–2020	BJ	93.28 ± 4.05b	16.03 ± 0.33b	160.08 ± 25.85a
	BN	127.04 ± 9.53a	21.83 ± 0.91a	180.33 ± 70.35a
2020–2021	BJ	108.72 ± 4.72b	20.14 ± 0.87b	201.14 ± 32.47a
	BN	163.99 ± 0.53a	25.29 ± 1.62a	232.77 ± 90.81a

BJ represents *Brassica juncea* oilseed rape green manure, and BN represents *Brassica napus* oilseed rape green manure. Different lowercase letters represent significant differences between different green manure treatments.

### 3.2 Effects of different treatments on soil physical and chemical properties

The results showed that compared with WT, the oilseed rape green manure returning treatment had a greater impact on soil organic matter and available potassium content (**Table 2**). The effect of oilseed rape green manure returning on soil organic matter and available potassium content reached a significant level. Compared with the WT, the oilseed rape green manure returning treatments showed a trend of increased contents of soil organic matter and available potassium, and the soil organic matter in BN was significantly increased by 17.16%. The soil available potassium content of BJ was significantly increased by 7.28%.

**Table 2:** Effects of oilseed rape green manure return on soil basic properties.

Treatments	pH	SOM g kg^−1^	AN mg kg^−1^	AK mg kg^−1^
BJ	5.70 ± 0.21a	20.59 ± 2.19ab	102.76 ± 10.53a	56.89 ± 5.52a
BN	5.66 ± 0.08a	21.78 ± 0.74a	109.92 ± 25.63a	55.07 ± 2.01ab
WT	5.76 ± 0.24a	18.59 ± 1.30b	108.72 ± 16.03a	53.03 ± 3.18b
NT	5.95 ± 0.27a	22.00 ± 3.04a	120.65 ± 13.13a	78.90 ± 10.30b

SOM is soil organic matter, AN is available nitrogen, AK is available potassium, BJ represents *Brassica juncea* oilseed rape green manure, BN represents *Brassica napus* oilseed rape green manure, WT represents winter fallow, and NT represents no tillage. Different lowercase letters represent significant differences between different treatments.

### 3.3 Effects of different treatments on phosphorus content in red soil

Compared with WT and NT, the soil total phosphorus content of BN was significantly increased by 5.26% and 17.65%, respectively, while the soil total phosphorus content of BJ was significantly increased by 13.73% compared with NT, but the differences did not reach a significant level compared with WT (**Table 3**). The content of soil particulate phosphorus in BJ and BN was significantly increased by 14.00% and 16.00% compared with the NT, respectively, but the difference between the two and the WT was not significant. Compared with WT and NT, the soil available phosphorus content of BJ and BN showed an increasing trend. The soil available phosphorus content of BJ was significantly higher than that of WT and NT by 11.12% and 13.01%, respectively, while in BN, it increased by 7.56% and 9.39%, respectively, without significant differences. Compared with WT and NT, the content of water-soluble inorganic phosphorus in the soil of BN significantly increased by 96.97% and 94.03%, respectively, while in BN, it increased by 13.64% and 11.94%, respectively, without significant differences. There was no significant difference between the contents of soil water-soluble total phosphorus and soil water-soluble organic phosphorus in all treatments.

**Table 3:** Effects of oilseed rape green manure return on components of soil P.

Treatments	STP g kg^−1^	TWSP mg kg^−1^	PP g kg^−1^	WSIP mg kg^−1^	WSOP mg kg^−1^	AP mg kg^−1^
BJ	0.58 ± 0.03ab	4.30 ± 1.97a	0.57 ± 0.03a	0.15 ± 0.03ab	4.22 ± 1.91a	9.99 ± 0.42a
BN	0.60 ± 0.02a	4.12 ± 0.64a	0.58 ± 0.03a	0.26 ± 0.11a	3.99 ± 0.84a	9.67 ± 0.66ab
WT	0.57 ± 0.03b	5.62 ± 2.22a	0.56 ± 0.03a	0.13 ± 0.03b	5.48 ± 1.61a	8.99 ± 0.79b
NT	0.51 ± 0.03c	4.32 ± 2.99a	0.50 ± 0.01b	0.13 ± 0.07b	4.22 ± 0.82a	8.84 ± 3.20b

STP is soil total phosphorus, TWSP is total water-soluble phosphorus, PP is particulate phosphorus, WSIP is water-soluble inorganic phosphorus, WSOP is water-soluble organic phosphorus, AP is available phosphorus, BJ represents *Brassica juncea* oilseed rape green manure, BN represents *Brassica napus* oilseed rape green manure, WT represents winter fallow, and NT represents no tillage. Different lowercase letters represent significant differences between different treatments.

**Figure 3:**
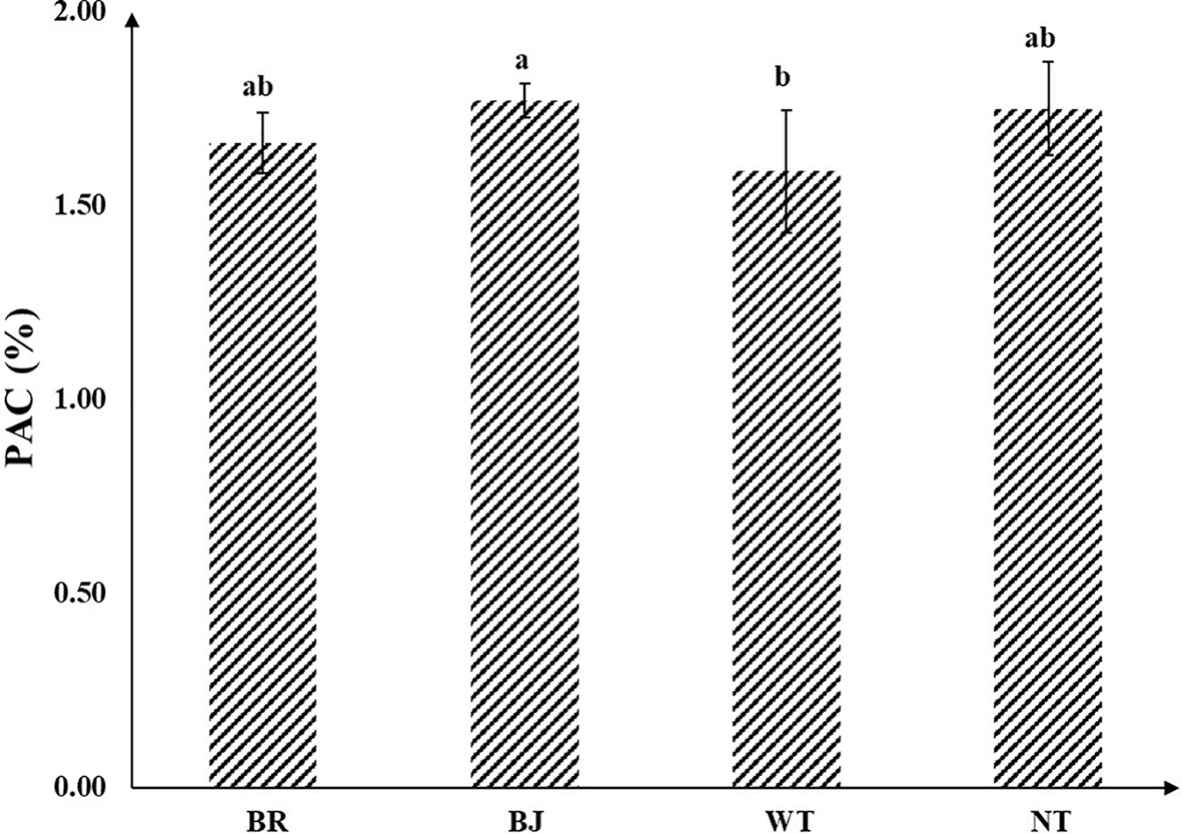
Effects of different treatments on soil PAC. PAC represents the activation coefficient of soil phosphorus, BJ represents mustard oilseed rape green manure, BN represents brassica oilseed rape green manure, WT represents winter fallow, and NT represents no tillage. Different lowercase letters represent significant differences between different treatments.

In this study, the phosphorus activation coefficients of experimental soil under different treatments were distributed between 1.49% and 1.77% (**Figure 3**). Compared with the WT, the phosphorus activation coefficient of BJ was significantly increased by 11.41%. Both BJ and BN showed no significant differences compared with NT.

### 3.4 Effects of different treatments on the growth and yield of subsequent crops

The results showed that compared with the WT, planting and returning oilseed rape green manure in winter could increase the tillering of rice to varying degrees (**Table 4**). The results of the two years showed that the tiller number of rice of BJ increased by 14.06% and 26.48%, respectively. The tiller number of rice in BN was increased by 4.29% and 21.62%, respectively, and the increase in BJ reached a significant level in 2020. The oilseed rape green manure returning to the field tended to increase the ear length of rice, but the difference compared with no return was not significant, and the effect on the grain number per panicle was not obvious in the two years reported here.

**Table 4:** Effects of oilseed rape green manure return on the growth of subsequent crop.

Treatments		Tiller number	Ear length cm	Grain number per spike	Yield kg ha^−1^	Biomass kg ha^−1^
2020	BJ	16.22 ± 1.86a	27.31 ± 0.98a	232 ± 22.70a	8,153 ± 1,085a	14,055 ± 1,092a
	BN	14.83 ± 2.55ab	27.22 ± 2.26a	226 ± 35.40a	8,198 ± 376a	14,233 ± 985a
	WT	14.22 ± 2.05b	26.26 ± 1.99a	247 ± 10.89a	7,727 ± 13b	13,936 ± 505a
2021	BJ	14.33 ± 2.06a	27.10 ± 1.09a	239 ± 24.93a	7,161 ± 40a	13,614 ± 439a
	BN	13.78 ± 2.64a	27.03 ± 2.05a	227 ± 38.93a	6,910 ± 13ab	13,364 ± 13a
	WT	11.33 ± 3.43a	26.13 ± 1.83a	248 ± 5.74a	6,077 ± 1,063b	11,716 ± 2,259a

BJ represents *Brassica juncea* oilseed rape green manure, BN represents *Brassica napus* oilseed rape green manure, and WT represents winter fallow. Different lowercase letters represent significant differences between different treatments.

The effects of different treatments on rice yield are shown in **Table 4**. The results showed that the measures of oilseed rape green manure returning to the field had a significant effect on rice grain yield. Compared with the WT treatment, the grain yield of rice in BJ was significantly increased by 5.51% and 17.84% in the respective two years, and the grain yield of rice in BN was significantly increased by 6.10% in 2020 and 13.71% in 2021 without a significant difference. Compared with WT, the biomass of rice (straw) in BJ and BN showed an increasing trend by 8.53% and 8.10% on average higher than that in the WT, respectively, but the difference was not significant.

**Figure 4:**
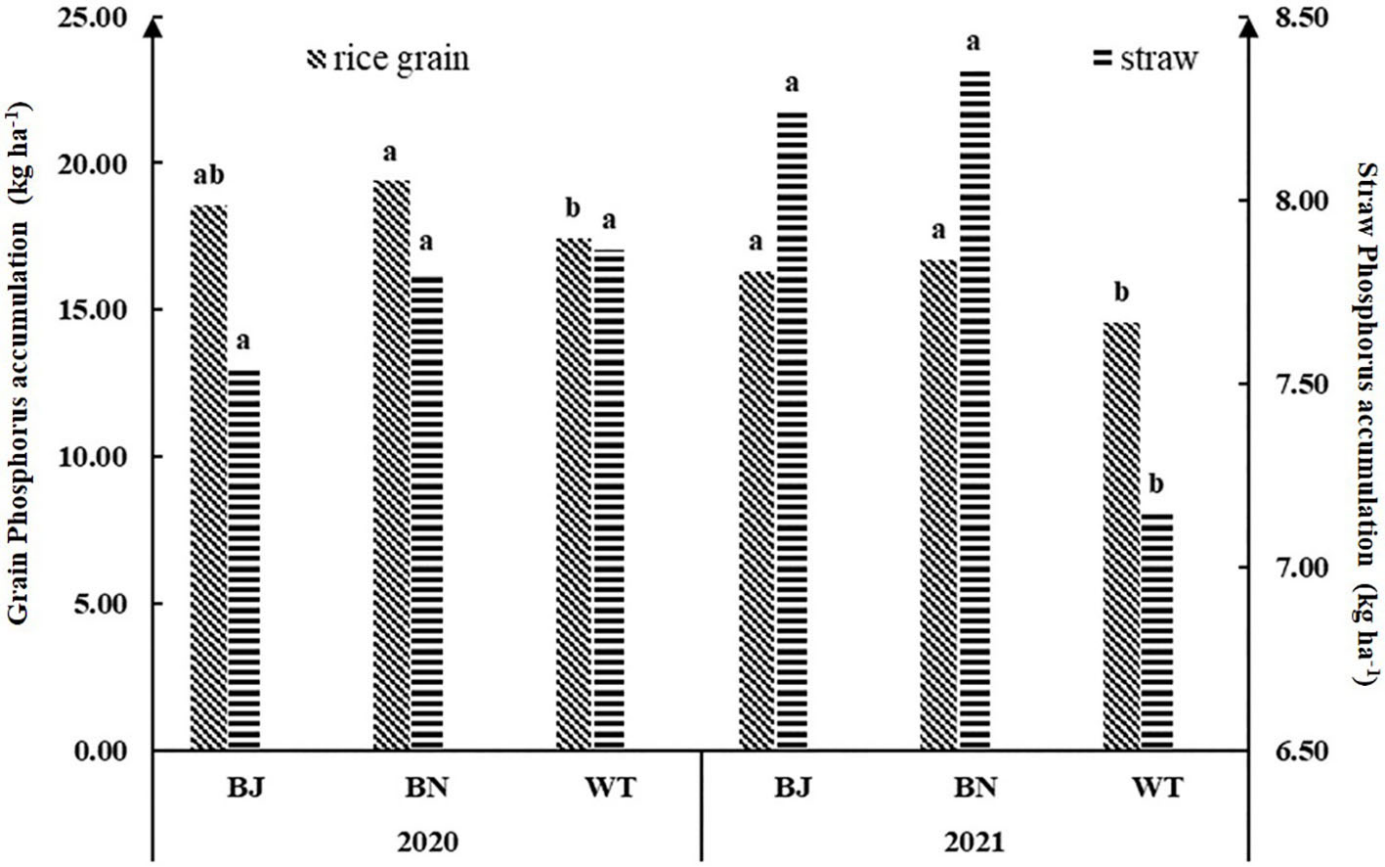
Effects of different treatments on the phosphorus accumulation of rice. BJ represents mustard oilseed rape green manure, BN represents brassica oilseed rape green manure, WT represents winter fallow, and NT represents no tillage. Different lowercase letters represent significant differences between different treatments.

The effects of different treatments on phosphorus accumulation in rice plants are shown in **Figure 4**. The results showed that the phosphorus accumulation in rice grain after the green manure returning treatments was higher than that of WT. The phosphorus accumulation in rice grain of BN was significantly higher than that of WT for both 2020 and 2021 by 11.21% and 14.82%, respectively. The phosphorus accumulation of rice grain treated with BJ increased by 6.56% and 11.98%, respectively, but only reached a significant level in 2021.

## 4 Discussion

### 4.1 Effects of different oilseed rape green manure returning on soil nutrients

After the oilseed rape green manure is returned to the field, the flowers and leaves decompose first, followed by the stems, with the roots last to decompose. This process effectively provides a continuous source of nutrients for the subsequent crops (Talgre et al., 2014; Gu et al., 2019). The decomposition rate of oilseed rape green manure was faster than leguminous green manure, with the decomposition rate of dry matter exceeding 80% 43 days after plowing (Gu et al., 2019). This could significantly increase the organic matter content of the topsoil, especially the increase of organic matter in the 0–20-cm soil layer, which could reach 161.5%–167.7% (Poeplau and Don, 2015). Therefore, the dry matter of the oilseed rape green manure plant itself can significantly increase the soil organic matter and nutrient content after decomposition (Fu et al., 2012). Zhou et al. (2023) found that the organic matter content of rice–rice–rape soil significantly increased by 7.3%–11.6% after 3 years of duplicated cultivation. In this experiment, both types of oilseed rape species in the rice–rape green manure rotation mode had a trend of increasing soil organic matter, and the BN significantly increased the soil organic matter content by 17.16%. This shows that different species of rape green manure returned to the field have different effects on soil organic matter. In the paddy field, the effect of BN on soil organic matter was more obvious than BJ. The higher biomass of BN was the main reason for this (**Figure 2**).

Studies have shown that planting oilseed rape can reduce the conversion of soil available phosphorus into O–P and make Ca–P in acidic soil more bioavailable and improve the effectiveness of soil phosphorus (Gu et al., 2023; Yuan et al., 2023). During the growth of oilseed rape green manure, root exudates can activate insoluble nutrients in the soil and increase the concentration of available nutrients in the soil, including phosphorus (Vives-Peris et al., 2020; Chen et al., 2023). In this study, the soil available phosphorus content of the two oilseed rape green manure treatments was higher than that of the WT, and the BJ was significantly higher than the WT by 11.12%. In addition, the returning of green manure of different oilseed rape species in this study also had a significant effect on available potassium in the red soil of the experimental site. The content of available potassium in the soil of BJ increased significantly by 7.28%, indicating that the oilseed rape green manure returning measures had a significant positive effect on the soil nutrient availability in the paddy field of the experimental site. Differences may be related to the differences between treatments in the green manure species, green manure decomposition process, and products of organic materials carried into the soil (Fu et al., 2012; Gu et al., 2019) during the growth of the oilseed rape green manures. The red soil of the experimental site contains a large amount of clay minerals, and both the growth and decomposition processes of oilseed rape green manure will promote the release of nutrients from clay minerals. More than 18.09 kg ha^−1^ of phosphorus on average and 170 kg ha^−1^ of potassium on average were accumulated during the oilseed rape green manure growth period (**Table 1**), and the decomposition rate can exceed 80% in a short time after returning to the field (Fu et al., 2012). Therefore, chemical fertilizers can be appropriately reduced after oilseed rape green manure returning to the field. At present, most of the related research regarding the substitution of chemical fertilizers by green manure has focused on the potential of Chinese milk vetch (Xie et al., 2022). There are few quantitative studies on the potential for reduction of chemical fertilizer application in rice production using oilseed rape green manure returned to the field, which is cheaper and better adapted to the requirements for rotation, justifying amplification in the future.

In addition, planting and returning oilseed rape green manure to the field will also have an effect on soil pH (Pimentel et al., 2023), which will lead directly to changes in bioavailability of soil phosphorus and other nutrients. The study of Li et al. (2012) showed that the application of tropical leguminous green manure affected the pH value of red soil, especially the green manure crops with high ash alkali content, which had a more obvious effect, increasing the pH value. However, no significant difference in soil pH value was observed in the oilseed rape green manure returning treatments in this study. This may be due to the low pH background value of red soil (5.34) in this study and the test time not being long enough, such that the effect of green manure on soil pH value was not significant.

### 4.2 Effects of different oilseed rape green manure returning on rice growth and yield

At present, there are few studies that focus on the effects of oilseed rape as green manure returned to the field on rice growth and yield in rice–oilseed rape rotation. Oilseed rape is a high-quality green manure with high fertilizer value for rice production due to its large biomass, easy decomposition, and low planting cost (Fu et al., 2012). Returning of oilseed rape green manure to the field has been found to have a significant effect on the production of rice. Researchers found that applying green manure in rice production mainly manifested in significantly increasing leaf area index, flag leaf length and leaf width, chlorophyll content, photosynthetic rate, dry matter accumulation, and effective tiller number (Zhou et al., 2023). The results of this study showed that planting and returning oilseed rape green manure in winter produced a trend of increasing the rice tiller numbers compared with WT. The rice tiller numbers of BJ were significantly increased by 43.16%, one of the main reasons for the significant effect of BJ on rice grain yield in this study.

Earlier studies found that the yield and quality of rice were higher when rice was rotated with oilseed rape rather than other crops, due to an increase in effective panicle number, grain number per panicle, and nitrogen accumulation (Meng et al., 2019; Zhang et al., 2020). The rice–oilseed rape green manure rotation not only produces high-quality green manure for rice season application but also activates the O–P in the red soil that cannot be directly absorbed and utilized by rice during the oilseed rape growth period. The oilseed rape green manure returning can regulate soil bulk density and soil texture and make the ratio of carbon to nitrogen in the soil conducive to the absorption of soil nutrients by rice (Yu et al., 2014). Confirming the results of earlier studies, this study found that compared with the WT, the oilseed rape green manure treatment showed a trend of increasing the yield of rice. In addition to increasing the effective panicle number and grain number per panicle, the incorporation of oilseed rape green manure can increase the photosynthetic rate and dry matter accumulation of rice, thereby increasing the yield of rice (Zhou et al., 2023; Wang et al., 2012). Green manure returning can effectively delay the senescence of rice leaves by promoting the activity of soil microorganisms, activating the enzyme activities related to nutrient absorption and utilization, and increasing the protease activity in the soil, thus promoting absorption and accumulation by rice plants (Tang et al., 2017). The results of this study showed that the return of oilseed rape green manure to the field had a significant effect on rice yield, and the grain yield of BJ and BN was significantly increased by 5.51% and 6.09%, respectively. Another recent study showed that the yield of early rice increased by 3.5% and that of late rice increased by 2.3% when oilseed rape was used as green manure in red soil paddy fields in rice–rice–rape rotation in southern China (Zhou et al., 2023), which was consistent with the experimental results of this study.

## 5 Conclusion

Rice–oilseed rape green manure rotation enhanced the phosphorus nutrients of red soil and promoted phosphorus availability. BN increased total phosphorus and water-soluble inorganic phosphorus content, while BJ increased water-soluble inorganic phosphorus content. The phosphorus activation coefficient of BJ increased, and the phosphorus accumulation of subsequent rice increased both in BN and BJ.

Rice–oilseed rape green manure rotation facilitated an increase in rice grain yield, likely due in part to the increase in phosphorus availability, as quantitatively elucidated in this study. These results present an excellent basis for the use of oilseed rape green manure rotation in substitution for harmful chemical fertilizers for productive rice cultivation in red soil areas.

## Data availability statement

The original contributions presented in the study are included in the article/supplementary material. Further inquiries can be directed to the corresponding authors.

## Author contributions

C-MG: Conceptualization, Data curation, Formal Analysis, Funding acquisition, Investigation, Methodology, Project administration, Resources, Software, Writing – original draft, Supervision, Validation, Visualization, Writing – review & editing. Y-SL: Formal Analysis, Investigation, Writing – review & editing. LY: Formal Analysis, Data curation, Software, Writing – review & editing. JD: Validation, Visualization, Writing – review & editing. WH: Validation, Visualization, Writing – review & editing. C-BY: Conceptualization, Methodology, Writing – review & editing. MB: Resources, Supervision, Writing – review & editing. XL: Conceptualization, Writing – review & editing. LQ: Conceptualization, Project administration, Resources, Supervision, Writing – review & editing.

## References

[B1] Asghar W. Kataoka R. (2022). Green manure incorporation accelerates enzyme activity, plant growth, and changes in the fungal community of soil. Arch. Microbiol. 204, 7. doi: 10.1007/s00203-021-02614-x 34870760

[B2] Bao S. D. (2000). Analysis of Soil Agronomy. 3rd ed (Beijing, China: China Agricultural Press), 34–231.

[B3] Chen S. Yang D. Wei Y. He L. Li Z. Yang S. (2023). Changes in soil phosphorus availability and microbial community structures in rhizospheres of oilseed rapes induced by intercropping with white lupins. Microorganisms 11, 326. doi: 10.3390/microorganisms11020326 36838291 PMC9959241

[B4] Fu T. D. Liang H. D. Zhou G. S. (2012). Advantages and development suggestions of rape green fertilizer in modern agriculture. China Agric. Tech. Ext. 28, 37–39. doi: 10.3969/j.issn.1002-381X.2012.08.020

[B5] Gu C. Huang W. Li Y. Li Y. Yu C. Dai J.. (2021). Green manure amendment can reduce nitrogen fertilizer application rates for oilseed rape in maize–oilseed rape rotation. plants-Basel 10, 2640. doi: 10.3390/plants10122640 34961111 PMC8704046

[B6] Gu C. Li Y. Xie L. Hu X. Liao X. Qin L. (2019). Analysis on application advantages of rapeseed as green manure. Soil Fertil. Sci. China 01, 180–183. doi: 10.11838/sfsc.1673-6257.18041

[B7] Gu C. Lv W. Liao X. Brooks M. Li Y. Yu C.. (2023). Green manure amendment increases soil phosphorus bioavailability and peanut absorption of phosphorus in red soil of south China. Agronomy-Basel 13, 2, 376. doi: 10.3390/agronomy13020376

[B8] Jing T. L. (2007). Spring rape green manure turn pressure interplanting potatoes. China Veg. 10, 52–53. doi: 10.3969/j.issn.1000-6346.2007.10.023

[B9] LeBlanc N. (2022). Green manures alter taxonomic and functional characteristics of soil bacterial communities. Microb. Ecol. 85, 684–697. doi: 10.1007/s00248-022-01975-0 35112152

[B10] Li P. Li Y. B. Xu L. Y. Zhang H. J. Shen X. S. Xu H. F.. (2021). Crop yield soil quality balance in double cropping in China’s upland by organic amendments: A meta-analysis. Geoderma 403, 115197. doi: 10.1016/j.geoderma.2021.115197

[B11] Li Y. Liu G. D. Zhang R. L. Huan H. F. Gao L. (2012). Dynamic effect on Latosol pH value after leguminous green manure application and relevant mechanism. Soils 44, 101–106. doi: 10.13758/j.cnki.tr.2012.01.014

[B12] Lyu H. Li Y. Wang Y. Wang P. Shang Y. Yang X.. (2024). Drive soil nitrogen transformation and improve crop nitrogen absorption and utilization - a review of green manure applications. Front. Plant Sci. 14. doi: 10.3389/fpls.2023.1305600 PMC10794358 38239220

[B13] Meng Y. Jin W. Dong Z. Wen Y. Liang F. Ding F.. (2019). Comparison of resource utilization efficiency and economic benefit of different paddyupland rotation systems in Jianghuai region. Chin. J. Ecol. 38, 3357–3365. doi: 10.13292/j.1000-4890.201911.029

[B14] Pimentel M. L. Reis I. M. S. Romano M. L. P. C. Castro J. S. de Vildoso C. I. A. Gasparin E.. (2023). Green manure, a sustainable strategy to improve soil quality: a case study in an oxisol from northern Brazil. Aust. J. Crop Sci. 17, 488–497. doi: 10.21475/ajcs.23.17.06.p3832

[B15] Poeplau C. Don A. (2015). Carbon sequestration in agricultural soils via cultivation of cover crops – a meta-analysis. Agric. Ecosyst. Environ. 200, 33–41. doi: 10.1016/j.agee.2014.10.024

[B16] Ren H. Lv H. Xu Q. Yao Z. Yao P. Zhao N.. (2024). Green manure provides growth benefits for soil mesofauna by promoting soil fertility in agroecosystems. Soil Till. Res. 238, 106006. doi: 10.1016/j.still.2024.106006

[B17] Srivastava J. N. Ghatak A. (2017). Biofumigation: A control method for the soil-borne diseases. Int. J. Plant Prot. 10, 453–460. doi: 10.15740/HAS/IJPP/10.2/453-460

[B18] Talgre L. Lauringson E. Roostalu H. Makke A. (2014). Phosphorus and potassium release during decomposition of roots and shoots of green manure crops. Biol. Agric. Hortic. 30, 264–271. doi: 10.1080/01448765.2014.953582

[B19] Tang H. M. Xiao X. P. Tang W. G. Wang K. Li C. Cheng K. K. (2017). Returning winter cover crop residue influences soil aggregation and humic substances under double-cropped rice fields. Rev. Bras. Cienc. Solo. 41, e0160488. doi: 10.1590/18069657rbcs20160488

[B20] Vives-Peris V. de Ollas C. Gómez-Cadenas A. Pérez-Clemente R. M. (2020). Root exudates: from plant to rhizosphere and beyond. Plant Cell Rep. 39, 3–17. doi: 10.1007/s00299-019-02447-5 31346716

[B21] Wang D. Y. Peng J. Xu C. M. Zhao F. Zhang X. F. (2012). Effects of rape straw manuring on soil fertility and rice growth. Chin. J. Rice Sci. 26(1), 85–91. doi: 10.3969/j.issn.1001-7216.2012.01.014

[B22] Xie J. Liang F. Xie J. J. Jiang G. Zhang X. Zhang Q. (2022). Yield variation characteristics of red paddy soil under long-term green manure cultivation and its influencing factors. Int. J. Environ. Res. Public Health 19, 2812. doi: 10.3390/ijerph19052812 35270509 PMC8910239

[B23] Xie Z. J. Tu S. X. Shah F. Xu C. X. Chen J. R. Han D.. (2016). Substitution of fertilizer-N by green manure improves the sustainability of yield in double-rice cropping system in south China. Field Crops Res. 188, 142–149. doi: 10.1016/j.fcr.2016.01.006

[B24] Yu Y. L. Xue L. H. Yang L. Z. (2014). Winter legumes in rice crop rotations reduces nitrogen loss, and improves rice yield and soil nitrogen supply. Agron. Sustain. Dev. 34, 633–640. doi: 10.1007/s13593-013-0173-6

[B25] Yuan B. Yu D. Hu A. Wang Y. Sun Y. Li C. (2023). Effects of green manure intercropping on soil nutrient content and bacterial community structure in litchi orchards in China. Front. Environ. Sci. 10. doi: 10.3389/fenvs.2022.1059800

[B26] Zhang S. T. Lu J. W. Cong R. H. Ren T. Li X. K. Liao S. P.. (2020). Effect of rapeseed rotation on the yield of next-stubble crops. Sci. Agric. Sin. 53, 2852–2858. doi: 10.3864/j.issn.0578-1752.2020.14.009

[B27] Zhou Q. Zhang P. Wang Z. Wang L. Wang S. Yang W.. (2023). Winter crop rotation intensification to increase rice yield, soil carbon, and microbial diversity. Heliyon 9, e12903. doi: 10.1016/j.heliyon.2023.e12903 36820165 PMC9938412

[B28] Zhu X. (2011). Effect of manure application on soil phosphorus components and phosphorus losses of farmland (Beijing, China: Chinese Academy of Agricultural Sciences).

